# Evaluation of the prevalence of musculoskeletal symptoms, presumptive diagnosis, medical care use, and sick leave among female school meal service workers

**DOI:** 10.1186/s40557-019-0281-0

**Published:** 2019-01-15

**Authors:** Young Hoon Moon, Young Joon Yang, Sang Yoon Do, Jae Yoon Kim, Chul Gab Lee, Hong Jae Chae, Soo Hyeon Kim, Han Soo Song

**Affiliations:** 1 Department of Occupational and Environmental Medicine, Chosun University Hospital, College of Medicine, Chosun University, Gwangju, Republic of Korea; 2 Department of Occupational and Environmental Medicine, Donggunsan Hospital, Gunsan, South Korea; 3 Department of Occupational and Environmental Medicine, KS hospital, Gwangju, South Korea

**Keywords:** School meal, Female, Musculoskeletal diseases

## Abstract

**Background:**

Most of the school meal service workers in Korea are middle-aged individuals. They have high workload, which increases their incidence of musculoskeletal disorders. This study aimed to evaluate the prevalence and risk factors of subjective musculoskeletal symptoms, presumptive diagnosis, medical care use, and sick leave among female school meal workers.

**Methods:**

We analyzed the results of musculoskeletal disease screening of 1581 female school meal workers. The screening consisted of self-administered questionnaire, history taking by occupational physicians, and physical examination. The prevalence of subjective musculoskeletal symptoms, presumptive diagnosis after initial examination by occupational physicians, use of medical care for more than 7 days, and sick leave due to musculoskeletal diseases during the past year were evaluated in this study. The relative risk of four outcome indicators of musculoskeletal disorders was compared with respect to potential factors, such as age, subjective physical loading, present illness, injury experience, and type of school, using log-binomial regression.

**Results:**

The prevalence of subjective musculoskeletal symptom was 79.6%; presumptive diagnosis, 47.6%; hospital visits over 7 days, 36.4%; and sick leave, 7.3%. The relative risk of musculoskeletal symptoms by age (≥50 years vs < 50 years) was 1.04 (95% confidence interval (CI): 1.00–1.09); presumptive diagnosis of musculoskeletal disease, 1.17 (95% CI: 1.06–1.30); hospital visits over 7 days, 1.26 (95% CI: 0.85–1.85); and sick leave, 1.17 (95% CI: 1.02–1.34). The relative risk of musculoskeletal symptoms due to subjective physical loading (very hard vs low) was 1.45 (95% CI: 1.33–1.58); presumptive diagnosis, 2.92 (95% CI: 2.25); hospital visits over 7 days, 1.91 (95% CI: 1.02–3.59); and sick leave, 2.11 (95% CI: 1.63–2.74).

**Conclusions:**

Subjective physical loading was a more important factor in musculoskeletal disorders than the age of female school meal workers.

## Background

Musculoskeletal disorders are the most common occupational diseases. In Korea, musculoskeletal disorders accounted for about 10% of all occupational diseases in the 1990s. In 2003, however, it accounted for 49.6% of all occupational diseases. Since 2006, it has consistently accounted for 60–70% of all occupational diseases. Of the 4947 workers, 76.64% of men and 20.36% of women were compensated for musculoskeletal diseases, according to workers’ compensation statistics from Korea Workers’ Compensation & Welfare Service in 2016 [1].

However, the burden of musculoskeletal disease in the general population is higher in women than in men. According to a study conducted in South Korea, back pain ranked first among the 30 major diseases in women and second in men based on the estimated disability-adjusted life years [2]. Another study conducted on a 20-to-64-year-old working population using the database of the National Health Insurance Service in South Korea reported that the total loss due to musculoskeletal diseases in men in 2008 was $16.74 billion and that of women was $32.09 billion. The productivity loss was $750 million for men and $1.53 billion for women [3].

South Korea’s public schools have been providing school meals for students since 2003. As a result, the number of school meal service workers had increased dramatically; most of them were middle-aged women. This job was easily accessible to individuals without a career because of similarities with housework. Hence, school meal workers have been exposed to musculoskeletal risk factors such as manual handling, repetitive motion, and awkward posture. Because older female workers performed high burden work, they had higher incidence of musculoskeletal diseases.

In this study, the musculoskeletal diseases of female school meal workers experiencing the highest level of physical burden were evaluated. Furthermore, details regarding the burden of musculoskeletal disease among female workers are limited. Some studies showed the prevalence and major site of musculoskeletal disorders among school meal service workers [4–8]. However, these studies did not provide sufficient information since they only used the musculoskeletal symptom questionnaire. Hence, this study aimed to investigate the presumptive diagnosis, medical care use, and sick leave rate of musculoskeletal diseases based on the physical examinations and history taking of occupational physicians. It also aimed to identify the risk factors for musculoskeletal disorders.

## Methods

### Participants and data collection

The Gwangju Metropolitan Office of Education (GMOE) conducted a screening program for prevention of musculoskeletal diseases among 1930 school meal employees from February to December 2016. The health examination of musculoskeletal disease was requested to three hospitals with occupational and environmental medicine departments. A total of 1581 (82%) individuals participated in this screening program. We obtained a written consent from all participants, which permits the use of collected data for preventive action. We retrospectively analyzed the data of the GMOE’s musculoskeletal disease screening program. This study was approved by the Institutional Review Board (Chosun 2018–05-015).

### Research tools

#### General characteristics and occupational characteristics

Variables were collected using the musculoskeletal symptom questionnaire developed by the Korea Occupational Safety and Health Agency (KOSHA) [9]. The variables consisted of job control, household work time, current illness, injury experience, current illness, injury experience, working hour, the number of meals per day, career of school meal service worker, type of school, and subjective physical loading. Job control is categorized based on whether it can control work speed and break time. Household work time refers to the average time spent performing certain tasks such as cooking, washing, cleaning, and taking care of children under 2 years old at home and is categorized by a 2-h cut-off. Current illnesses mean diagnosis of diseases such as rheumatoid arthritis, diabetes, lupus disease, gout, and alcoholism by physicians. Moreover, they developed hand, finger, wrist, arm, elbow, shoulder, neck, waist, leg, and feet injuries due to exercise-related accidents, traffic accidents, falls, and crash. Subjective physical loading was assessed on a five-point scale (very low, low, slightly hard, hard, and very hard) based on the physical burden felt for current work and was categorized into three levels by combining “very low,” “low,” and “slightly hard” into “low.”

### Four outcome indicators of musculoskeletal diseases

To investigate the characteristics of musculoskeletal diseases in female school meal workers, the four outcome indicators were categorized as follows: subjective musculoskeletal symptoms, presumptive diagnosis, the rate of hospital visits over 7 days during the past 12 months, and the rate of sick leave during the past 12 months. The subjective musculoskeletal symptoms were defined as pain that developed more than once per month or pain that lasted more than 1 week, which was assessed using the musculoskeletal symptoms questionnaire from KOSHA [4]. The presumptive diagnosis was defined as being diagnosed and treated as a musculoskeletal disease by a doctor or having severe pain and objective signs upon physical examination. Severe pain was characterized by difficulty sleeping and restricted work performance or limited performance of daily activities. Pain severity was assessed by the doctor through history taking. Objective signs on physical examination were defined as positive response in provocation test, tenderness, and limited range of joint motion.

The detailed diagnosis of musculoskeletal disorders was made based on the following criteria. Myofascial syndrome has pain on the affected muscle or referred pain, tenderness, taut band, and pain during contraction. Finger joint osteoarthritis has joint stiffness or pain during joint motion and limited range of joint motion, Heberden’s node, Bouchard’s node, and joint swelling. Elbow epicondylitis has pain and tenderness in the medial or lateral epicondylar area without neurological symptoms and symptom induced by resistance against bending and extension of the wrist. Rotator cuff syndrome has no abnormal sensation in the corresponding shoulder area, but the pain is present with a positive sign in empty or full can test, impinge (Neer sign and Hawkins-Kennedy test) test, resisted internal rotation test, external rotation test, or lift-off test. Carpal tunnel syndrome is marked by intermittent sensory disorders or pain in the first, second, third, and fourth fingers and pain in the wrist and palms or radiating pains in the proximal part of the wrist. Phalen’s test, Tinel’s test (percussion), and wrist compression test revealed positive results. Finger tenosynovitis is marked by pain when moving the tendon and tenderness when palpating the tendon. Lumbar radiculopathy is characterized by leg pain, numbness, difficulty in walking, positive straight leg raising test, positive Lasegue sign, sensory changes in the specific site, or decline in motor function. Cervical radiculopathy is marked by intermittent pain and neck stiffness. With regard to head movement, an abnormal sensation or pain is felt that stretches from the neck to the upper limb. A pain is felt in the upper extremity during active or passive neck rotation and the result of Spurling test is positive. Knee osteoarthritis is characterized by knee pain in individuals older than 40 years of age, morning stiffness in the knees with bone spurs quadriceps muscle atrophy, or presence of varus deformity. These diagnostic criteria were shared by occupational and environmental physicians who participated in the screening.

To investigate the rate of medical care use and sick leave among workers with musculoskeletal diseases, the following question was asked: “Have you ever been absent or treated for musculoskeletal (joints, muscles, ligaments, tendons, and nerves) pain during the past 12 months (excluding accidental injuries, visits to the hospital for health checkups, and absence due to personal issues)?” Hence, we categorized medical care use and sick leave as follows: more than 7 days of medical care use and more than 1 day of sick leave.

### Statistical analysis

The univariate and multivariate analysis was conducted to examine the relationship between the four outcome indicators and potential risk factors such as job control, household work time, current illness, injury experience, working hour, number of meals per day, type of school, and subjective physical loading. We compared the relative risk of each outcome indicators by log-binomial regression analysis using SAS 9.3 (SAS Institute Inc.).

## Results

### General characteristics and work-related factors

Table 1 shows the general characteristics and work-related factors of the study population. The mean age was 51.1 years (standard deviation: 5.4). The rate of subjective physical loading was only 16.0%. The rates of present illness and injury experience were 10.4 and 25.0%, respectively.

**Table 1:** Characteristics of the study participants

Variables		Frequency	%
Age (years)	< 50	591	37.4
	≥50	990	62.6
Subjective physical loading	Low or slightly hard	252	16.0
	Hard	650	41.2
	Very hard	676	42.8
Present illness	No	1417	89.6
	Yes	164	10.4
Injury experience	No	1186	75.0
	Yes	395	25.0
Frequency of meal service	1	1179	74.6
	2–3	402	25.4
Career (years)	~ 9	635	40.3
	10~19	864	54.9
	≥20	75	4.8
Type of job	Chefs (manager)	263	16.6
	Cooks or assistants	1318	83.4
Job control	Yes	591	37.6
	No	982	62.4
Household work (hours)	< 2	1052	66.7
	≥2	526	33.3
Working hour	< 48	1285	81.3
	≥48	296	18.7
Type of school	High	467	29.5
	Middle	358	22.6
	Elementary	756	47.8
Total		1581	100.0

### Prevalence of the four outcome indicators according to major variables

Table 2 shows that the prevalence of subjective musculoskeletal symptoms was 79.6%, presumptive diagnosis was 47.6%, medical care use over 7 days was 36.4%, and sick leave by musculoskeletal disease was 7.3%. The four outcome indicators showed differences according to age, subjective physical loading, presence of present illness, injury experience, number of meals per day, the career of school meals service, and the type of school.

**Table 2:** Prevalence of musculoskeletal disorder related outcomes according to major variables

Variables		Subjective symptoms, %	Presumptive diagnosis, %	Medical care use, %	Sick leave, %
Age (years)	< 50	75.5 *	42.5*	32.5*	5.9*
	≥50	82.0	50.7	38.8	8.1
Physical loading	Low	46.8 *	19.4*	20.2*	4.4*
	Hard	79.8	47.0	33.5	6.2
	Very hard	91.7	58.7	45.3	9.5
Present illness	No	78.5*	44.6*	34.9*	6.9
	Yes	89.0	73.8	49.4	10.4
Injury experience	No	77.2*	45.8*	34.1*	6.2*
	Yes	86.6	53.0	43.3	10.6
Frequency of meal service	1	81.5*	48.8	38.6*	8.1*
	2-3	73.9	44.0	30.1	4.7
Career (years)	~ 9	73.9*	42.4*	30.9*	6.3
	10~19	83.2	51.2	40.0	7.6
	≥20	89.3	54.7	45.3	12.0
Type of job	Chefs (manager)	79.8	47.9	36.9	8.4
	Cooks or assistants	79.5	47.5	36.3	7.1
Job control	Yes	78.2	44.2*	37.1	6.4
	No	80.4	49.5	36.0	7.8
Household work (hours)	< 2	78.6	48.0	35.5	6.7
	≥2	81.7	47.0	38.4	8.6
Working hour	< 48	80.2	48.8	37.5	7.5
	≥48	77.0	42.6	31.8	6.4
Type of school	High	74.9*	43.5	31.0*	5.6
	Middle	81.3	49.0	38.5	6.1
	Elementary	81.6	49.5	38.8	8.9
Total		79.6	47.6	36.4	7.3

*p < 0.05 by chi-squared test

### Prevalence of presumptive diagnosis of doctors

Table 3 shows the details of presumptive diagnosis of doctors. The most frequent diagnosis was myofascial syndrome (241 patients, 15.2%), followed by finger joint osteoarthritis (233 patients, 14.7%), elbow epicondylitis (171 patients, 10.8%), rotator cuff syndrome (141 patients, 8.9%), carpal tunnel syndrome (100 patients, 6.3%), and finger tenosynovitis (62 patients, 3.9%). There were significant differences in the prevalence of finger joint osteoarthritis and rotator cuff syndrome by age. Subjective physical loading showed a significant relationship with myofascial syndrome, finger joint osteoarthritis, elbow epicondylitis, rotator cuff syndrome, carpal tunnel syndrome, finger tenosynovitis, and lumbar radiculopathy.

**Table 3:** Prevalence of musculoskeletal disorders by presumptive diagnosis of doctors

Presumptive diagnosis	Total (of 1581)		Age, n (%)				Subjective physical loading, n (%)					
			< 50 years		≥50 years		Low		Hard		Very hard	
Myofascial syndrome	241	(15.2)	91	(15.4)	150	(15.2)	11	(4.4)	99	(15.2)	131	(19.4)*
Finger joint osteoarthritis	233	(14.7)	62	(10.5)	171	(17.3)	17	(6.7)	80	(12.3)	136	(20.1)*
Elbow epicondylitis	171	(10.8)	58	(9.8)	113	(11.4)	3	(1.2)	71	(10.9)	97	(14.3)*
Rotator cuff syndrome	141	(8.9)	39	(6.6)	102	(10.3)	67	(9.9)	62	(9.5)	11	(4.4)*
Carpal tunnel syndrome	100	(6.3)	39	(6.6)	61	(6.2)	3	(1.2)	38	(5.8)	59	(8.7)*
Finger tenosynovitis	62	(3.9)	28	(4.7)	34	(3.4)	2	(0.8)	34	(5.2)	26	(3.8)
Lumbar radiculopathy	34	(2.2)	10	(1.7)	24	(2.4)	1	(0.4)	11	(1.7)	22	(3.3)
Cervical radiculopathy	29	(1.8)	8	(1.4)	21	(2.1)	3	(1.2)	10	(1.5)	16	(2.4)
Knee osteoarthritis	24	(1.5)	5	(0.8)	19	(1.9)	1	(0.4)	11	(1.7)	12	(1.8)

*p < 0.01 by chi-squared test

### Relationship between major variables and outcome indicators

Table 4 shows the relative risk of four outcome indicators by age, subjective physical loading, present illness, and injury experience. In particular, the relative risks for subjective musculoskeletal symptoms, presumptive diagnosis, and medical care use among workers who had very hard physical loading were 1.45 (95% confidence interval (CI): 1.33–1.58), 2.92 (95% CI: 2.25–3.78), 1.91 (95% CI: 1.02–3.59), and 2.11 (95% CI: 1.63–2.74) compared with those who had low physical loading.

**Table 4:** Relative risk of four outcome indicators of musculoskeletal disorders according to major variables

Variables		Subjective symptoms, %	Presumptive diagnosis by physician, %	Medical care use (≥7 days/year), %	Sick leave by musculoskeletal diseases%
		RR (95% CI)	RR (95% CI)	RR (95% CI)	RR (95% CI)
Age (years)	< 50	1.00	1.00	1.00	1.00
	≥50	1.04 (1.00–1.09)	1.17 (1.06–1.30)	1.26 (0.85–1.85)	1.17 (1.02–1.34)
Subjective Physical loading	Low	1.00	1.00	1.00	1.00
	Hard	1.32 (1.21–1.44)	2.39 (1.84–3.11)	1.32 (0.68–2.53)	1.59 (1.21–2.08)
	Very hard	1.45 (1.33–1.58)	2.92 (2.25–3.78)	1.91 (1.02–3.59)	2.11 (1.63–2.74)
Present illness	No	1.00	1.00	1.00	1.00
	Yes	1.03 (0.98–1.08)	1.51 (1.37–1.66)	1.28 (0.78–2.08)	1.26 (1.06–1.48)
Injury experience	No	1.00	1.00	1.00	1.00
	Yes	1.04 (0.99–1.08)	1.05 (0.95–1.15)	1.60 (1.11–2.30)	1.16 (1.02–1.33)
Type of school	High	1.00	1.00	1.00	1.00
	Middle	1.04 (0.98–1.10)	1.09 (0.96–1.24)	1.08 (0.62–1.88)	1.23 (1.02–1.47)
	Elementary	1.00 (0.96–1.05)	0.99 (0.89–1.11)	1.46 (0.94–2.26)	1.16 (0.99–1.36)

By log-binomial regression. Adjusted by age, subjective physical loading, present illness, injury experience, and type of school

## Discussion

According to the results of this study, the rate of musculoskeletal symptoms among school meal workers was very high (79.5%) but relatively low compared with those reported in previous studies. Compared with the results of other studies that applied the same KOSHA criteria for musculoskeletal symptoms, the rate of subjective musculoskeletal symptom was 91.4% in a study of 326 female food service workers in Nowon-gu, Seoul [4]. In the study of 1513 female food service workers in Gangwon-do in 2012, the rate of subjective musculoskeletal symptoms was 93.4% [5]. In a study of 891 female school meal workers in Seoul in 2012, the rate of subjective musculoskeletal symptoms was 89.0% [6]. The proportion of musculoskeletal symptoms reported in an unpublished study of school meal service workers in Gwangju metropolitan city was 89.8% in 2013 [8]. It is unclear whether the relatively low rate of musculoskeletal symptoms rate in this study represents a reduction in actual musculoskeletal disease. However, since the GMOE has promoted preventive education and improvement of food service facilities, the effectiveness of these efforts may have been reflected. Nevertheless, compared with other occupations, the rate of musculoskeletal symptom among school meal workers is very high. For example, the rate of musculoskeletal symptoms among melon-cultivating farmers was 75.2% [9], and the rate of musculoskeletal symptoms among automobile engine assemblers was 42.2% [10].

According to presumptive diagnosis by physicians, myofascial pain syndrome was most common musculoskeletal disorder among school meal workers. This disorder can affect any of the skeletal muscles in the body and the prevalence varies by physician [11, 12]. The prevalence of musculoskeletal diseases affecting the upper limb was relatively higher than that in other body parts. In particular, finger- and wrist-related diseases were common. This result is similar with those reported in other studies [4, 5]. School meal service consisted of several tasks such as preprocessing, cooking, feeding, washing dishes, and cleaning the kitchen accompanied by repetitive movements, uncomfortable postures, and excessive use of force of the upper limbs. The use of relatively heavy metal plates and cooking utensils, or high intensity of kitchen cleaning for hygiene control is considered to be an additional risk factor.

In this study, the risk of musculoskeletal symptoms, presumptive diagnosis, and sick leave was significantly higher in patients older than 50 years of age, but the relative risks of musculoskeletal symptoms and presumptive diagnosis were 1.04 and 1.17, respectively. There were significant differences in the prevalence of finger joint osteoarthritis and rotator cuff syndrome by age. This result is similar with previous studies [13–15]. There are few cases of knee osteoarthritis in this study to determine whether a result is statistically significant.

By contrast, the relative risks of musculoskeletal symptoms and presumptive diagnosis by subjective physical loading were 1.45 and 2.92, respectively. According to a previous study conducted on 7100 food service workers in Japan, a large number of meals per person, insufficient rest in the morning, poor kitchen environment, and inadequate height of the countertop were significant risk factors of musculoskeletal disorders [16]. A 2-year prospective study of 385 employees in a community restaurant in Finland showed that high physical workloads play a major role in predicting the occurrence of multisite musculoskeletal pain. This study has shown that higher physical workloads have a greater impact on musculoskeletal pain incidence than individual factors such as obesity and smoking [17]. In a survey of 114 elementary school meal workers showed that age, job stress, and presence of lunchroom were not significant risk factors for musculoskeletal symptoms. However, when the number of meals per worker was over 150, the odds ratio adjusted by age, job stress, and presence of lunchroom was 4.67 (95% CI: 1.04–21.0) [8]. In a study of school meal workers in Seoul, it was concluded that job demands and stress due to physical environment were associated with musculoskeletal symptoms, with odds ratios of 3.3 and 2.5, respectively [6]. Therefore, in the case of school meal workers, the physical labor intensity is generally the most important risk factor for musculoskeletal disorders than age. The groups that responded “very high” to the physical loading showed a lower prevalence rate of rotator cuff syndrome than the group that responded “low or high”. We supposed that this phenomenon is a survivor effect, because patients suffering from this disease are no longer able to perform high-level burden work.

According to the results of this study, the prevalence of medical care use and sick leave are low considering high prevalence of musculoskeletal disorder. We supposed through interviews with school meal service worker that this phenomenon is due to a lack of alternative labor. They complained that their co-workers should have an excessive workload if they have sick leave.

This study had some limitations. First, physical loading was evaluated using a self-reported questionnaire. The number of meals per worker was widely used to identify physical loading. However, this indicator does not reflect the physical loading accurately, because food service environment varied depending on the modernization or layout of school’s kitchen, the age of the students served with meals, and additional tasks such as food preparation and cleaning. Rather, subjective physical loading can reflect this situation comprehensively. Second, there is likely to be a bias in the presumptive diagnosis of musculoskeletal disorders in this study. The six occupational physicians participating in musculoskeletal screening may have had a different experience regarding the diagnosis of musculoskeletal disorders and may have a different understanding of the diagnostic criteria. However, since most of the diagnostic criteria for musculoskeletal diseases are not clear, epidemiologic studies of musculoskeletal disorders can only be made by operational definition. In this study, the presumptive diagnosis of the physician was based on the necessity of disturbance of daily life due to pain and discomfort and the existence of objective signs. This type of diagnosis complements the limitations of subjective symptom reporting. Third, the relationship shown in this study seemed to be underestimated or overestimated due to selection bias. Therefore, other evidence is needed to determine the causality between related factors and musculoskeletal disorders. However, musculoskeletal diseases have a long prevalence duration, and there is short latency period between the exposure to the harmful factors and the onset of the disease; hence, even a cross-sectional study can be one of evidence for judging the causal relationship.

The strength of this study was the large number of participants. In addition to the subjective symptoms assessed using a questionnaire, the results were obtained through doctors’ surveys and physical examinations, as well as assessing the burden of diseases such as hospital use and sick leave.

## Conclusion

In this study, the musculoskeletal symptoms were investigated using questionnaires and by conducting physical examinations of school meal workers. The rate of musculoskeletal symptoms was 79.6%; musculoskeletal patients, 47.6%; medical care use for 7 days or more, 36.4%; and sick leave, 7.3%. It is confirmed that the level of subjective physical loading is an important factor in developing musculoskeletal diseases rather than the age of school meal workers.

These results suggest that it is important to reduce physical burden, especially the upper limb physical burden for prevention of musculoskeletal diseases among food service workers.

## Abbreviations

CI: Confidence interval
COMWEL: Korea Workers’ Compensation & Welfare Service
GMOE: Gwangju Metropolitan Office of Education
KOSHA: Korea occupational safety and Health agency
